# Molecular typing and characterization of *Staphylococcus aureus* isolates from burn wound infections in Fujian, China

**DOI:** 10.3389/fmicb.2023.1236497

**Published:** 2023-09-20

**Authors:** Xiaolan Hong, Shaobo Zhou, Xubo Dai, Dandan Xie, Yuanyuan Cai, Guimei Zhao, Bin Li

**Affiliations:** ^1^Department of Clinical Laboratory, Fujian Medical University Union Hospital, Fujian, China; ^2^Department of Clinical Laboratory, The 910 Hospital of Joint Logistics Support Force of Chinese People's Liberation Army, Fujian, China

**Keywords:** *S. aureus* virulence genes, molecular typing, antimicrobial susceptibility, burn wound infection, biofilm-forming isolates

## Abstract

**Background:**

*Staphylococcus aureus* (*S. aureus*) is the most common causative agent of burn wound infection, that often leads to high morbidity and mortality. However, there is not enough knowledge about the molecular epidemiology and antimicrobial susceptibility of *S. aureus* isolates from burn wound infections in Fujian, China.

**Methods:**

Between 2016 and 2021, 90 *S. aureus* isolates were collected from burn wound infections in Fujian, China, including 59 methicillin-resistant (MRSA) strains and 31 methicillin-susceptible (MSSA) strains. These were investigated for molecular characteristics, virulence genes, biofilms, and antimicrobial susceptibility. All the isolates were genotyped by multilocus sequence typing (MLST), *spa* typing, *agr* typing, and SCC*mec* typing. Conventional PCR was performed for the detection of virulence genes. Biofilm formation capacity was assessed by tissue culture plate assay (TCP). The antimicrobial susceptibility of the isolates was evaluated using the dilution method.

**Results:**

In total, 37 sequence types (ST) and 34 Staphylococcal protein A (*spa*) types (including a new type named *spa*-t20720) were identified based on multilocus sequence typing (MLST) and *spa* typing, respectively. CC8-ST239-t030-*agr*I-SCC*mec*III (57.6%,34/59) and CC7-ST7-t091-*agr*I (16.1%, 5/31) represented the main clone of MRSA and MSSA isolates, respectively. Antibiotic susceptibility testing identified a significant difference in resistance rates between ST239 and non-ST239 isolates (*p* < 0.05). Twelve virulence genes were detected, of which the most common were *icaA* and *icaD* (both 100%), followed by *icaB* and *icaC* (both 96.7%), *icaR* (95.6%), *lukED* (81.1%), *lukAB* (62.2%), *pvl* (50%), *hlgBC* (26.7%), and *eta* (4.4%). Moreover, *lukAB*, *hlgBC*, *agr*I, and *agr*III were significantly correlated with burn severity (*p* < 0.05). MRSA isolates were less likely, compared with MSSA isolates, to carry *pvl*, *lukAB,* and *hlgBC* (*p* < 0.05). A new spa type, t20720, was identified that contains *pvl*, *lukED*, *lukAB*, *hlgBC*, *icaA*, *icaB*, *icaC*, *icaD,* and *icaR* genes and has strong biofilm formation ability.

**Conclusion:**

CC8-ST239-t030-*agr*I-SCC*mec*III and CC7-ST-7-t091-*agr*I were the prevalent molecular signatures of MRSA and MSSA isolates from burn wound infections in Fujian, China, respectively. The newly identified *spa*-t20720 isolate, which carries a wide range of virulence genes and has strong biofilm formation ability, requires special clinical attention.

## Introduction

In China, millions of burn injuries occur each year, and over 70% of mortality in burn wards occurs due to infection (Kalligeros et al., 2019). A study on the distribution of pathogens in burn patients in China from 2006 to 2019 showed that 87.98% (7,003/7960) of burn wounds contained pathogenic strains, and *S. aureus* was the most commonly isolated pathogen (Chen et al., 2021). Notably, burn wound infection is an important source of systemic infection. *S. aureus,* through burn wounds, can reach blood, organs, and tissues producing toxins causing systemic clinical symptoms and even septic shock, which has a poor prognosis and high mortality rate (Tong et al., 2015). *S. aureus* can also form biofilms causing persistent infections that delay wound healing (Sabirova et al., 2015).

*Staphylococcus aureus* isolates, with genome sequence differences of up to 22%, have high genetic diversity and rich phenotypes (Cuteri et al., 2004). Typically, the molecular characteristics of *S. aureus* isolates differ depending on the region. ST59 is the predominant strain in China (Wang et al., 2022), while ST8 and ST121 are common in the USA (O'Hara et al., 2012; Rao et al., 2015). Meanwhile, the molecular epidemiology of *S. aureus* also varies with different types of infections. Wang et al. reported that in pediatric patients with bloodstream infections, major methicillin-susceptible *S. aureus* (MSSA) clones belonged to ST188 and ST7 types, while the predominant methicillin-resistant *S. aureus* (MRSA) clone was ST59 (Wang et al., 2018).

There is limited information on the epidemiological characteristics of *S. aureus* isolates from burn wound infections worldwide. ST250-t928 was the major MRSA clone in a Ghanaian burn unit (Amissah et al., 2017), whereas ST239-SCC*mec* III/t037 was the major MRSA clone from burn wound patients in Iran (Goudarzi et al., 2017). Other studies on burn wounds have focused on the distribution and drug resistance analysis of pathogenic bacteria (Chen et al., 2021; Ozlu and Basaran, 2022). Therefore, we isolated *S. aureus* isolates from burn patients in the Fujian province (China) to better understand their epidemiological trends and molecular characteristics. Our data may help manage clinical *S. aureus* burn infections in this geographical area.

## Methods

### *Staphylococcus aureus* isolates

In total, 90 non-duplicated *S. aureus* clinical isolates were obtained from burn wound infections from five hospitals between 2016 and 2021, which were located in five different areas of Fujian province, including Quanzhou (the 910 Hospital of Joint Logistics Support Force of Chinese People’s Liberation Army), Zhangzhou (the 909 Hospital of Joint Logistics Support Force of Chinese People’s Liberation Army), Xiamen (the 73rd Army Hospital of the Army), Fuzhou (Fujian Medical University Union Hospital), and Ningde (Mindong Hospital of Ningde). This retrospective study examined data and bacterial strains from the admitted patients who met the following conditions. First, they had no apparent infection symptoms during the incubation period. Second, the infection occurred 48 h after admission. Third, the clinical specimens collected from burn wounds were identified as MRSA or MSSA in the laboratory. The strains were identified by VITEK 2 Compact system and GP Test Kit (bioMerieux, France).

### Burn severity grading

Burn severity grading (BSG) was performed based on the judgment criteria of the 1970 China Burn Conference-*Mild burns*: a second-degree burn with a total area of <10%; *Moderate burns*: a second-degree burn with a total area of 11–30% or a third-degree burn with an area of ≤9%; *Severe burn*: a second-degree burn with a total area of 31–50% or a third-degree burn with an area of 10–20%; *Extra-severe burn*: a patient with a total burn area of ≥50% or a third-degree burn with an area of ≥20%.

### Antimicrobial susceptibility testing

Antimicrobial susceptibility testing was performed using the VITEK 2 Compact system and GP-67 Test Kit (bioMerieux, France). Fourteen antibiotics, including penicillin (PEN), oxacillin (OXA), erythromycin (ERY), clindamycin (CLI), tetracycline (TET), gentamicin (GEN), ciprofloxacin (CIP), levofloxacin (LEV), rifampicin (RIF), trimethoprim/sulfamethoxazole (SXT), quinupristin/dalfopristin (QD), linezolid (LZD), tigecycline (TCG), and vancomycin (VAN), were used. *S. aureus* ATCC 25923 and 29213 strains were used as quality control strains, and the results were interpreted following the Clinical and Laboratory Standards Institute (CLSI) guidelines (CLSIM100, 2022, 32nd edition).

### *Staphylococcus aureus* protein A(*spa*) typing

Genomic DNA from *S. aureus* isolates was extracted using the Ezup Column Bacteria Genomic DNA Purification Kit (Sangon Biotech, Shanghai) and stored at −20°C. The amplification of the *spa* repeat region was carried out as described in previous studies (Uhlén et al., 1984). The resulting amplicons were sent to Sangon Biotech (Shanghai, China) for DNA sequencing, followed by analysis using the Ridom web server.^1^ Sequences were submitted to the Ridom web server (see footnote 1) to distinguish whether those strains were new *spa* types. When sequences could not be categorized, isolates will be defined as non-typable (NT). Primers were listed in Supplementary File S1.

### Multilocus sequence typing

Multilocus sequence typing (MLST) was carried out following the previously described protocol. Seven housekeeping genes (*arc*, *aro*, *glp*, *gmk*, *pta*, *tpi*, and *yqil*) were PCR amplified (He et al., 2018). The resulting amplicons were sent to Sangon Biotech (Shanghai, China) for DNA sequencing, and the results were compared with known alleles in the MLST database^2^ for MLST of the isolates. Related STs were clustered using eBURST, and evolutionary trees were constructed in the MEGA6 software. Primers were listed in Supplementary File S1.

### *agr* typing

The *agr* typing of isolates was performed by multiplex PCR following the previously described protocol (He et al., 2018), and in total, four types (I-IV) were examined. *S. aureus* isolates that could not be categorized into any known type were defined as NT. Primers were listed in Supplementary File S1.

### *Staphylococcus* chromosomal cassette *mec* (SCC*mec*) typing

MRSA isolates were subjected to SCC*mec* typing (I, II, III, IVa, IVb, IVc, IVd, and V) by multiplex PCR, as described previously (He et al., 2018). All isolates were also examined for the *mec*A gene. MRSA isolates that could not be classified were defined as NT. Primers were listed in Supplementary File S1.

### Detection of virulence genes

We detected 12 virulence genes (including 5 leukotoxin genes, *pvl*, *lukED*, *lukAB*, *hlgBC*) (He et al., 2018), and *lukMF*′ (Jarraud et al., 2002); 2 exfoliative toxin genes, *eta* and *etb* (Li et al., 2018); 5 *ica* operon genes, *icaA*, *icaB*, *icaC*, *icaD*, and *icaR* (Mahmoudi et al., 2019) were detected by PCR as described previously. Primers were listed in Supplementary File S1.

### Detection of biofilm formation ability

Biofilm formation ability was examined using the tissue culture plate assay (TCP) as described previously with some modifications (Gad et al., 2009). Strains, stored at −80°C, were inoculated on a Columbia Blood Agar plate for overnight culture at 35°C. Then, bacteria were selected and inoculated in fresh Trypticase Soy Broth (TSB) and cultured at 35°C for 24 h. The bacterial solution was diluted to 1.5 × 10^8^ CFU/mL with TSB. 200 μl/well of the respective diluted bacterial solution was added to a sterile 96-well cell culture plate; each strain was plated in triplicate. Equal amounts of TSB broth were added to the negative control wells. *S. aureus* ATCC29213 was used as the positive control strain. The culture solution was discarded from the well, and the wells were gently washed with sterile phosphate buffer saline (PBS; pH 7.2) three times to remove any floating bacteria. Next, 200 μL methanol was added to each well for fixation of bacterial cells for 15 min. After discarding the methanol and air drying, 200 μl of 1% crystal violet was added to the respective well and incubated for 30 min, followed by the wells washing (thrice) with sterile PBS. Finally, 200 μl 95% ethanol was added to respective wells to dissolve the dye crystals, and the plate optical density was recorded at 595 nm. The degree of biofilm production was scored as follows: absent (OD < ODc), weak (ODc < OD < 1.5 × ODc), moderate (1.5 × ODc < OD < 2 × ODc), and strong (OD > 2 × ODc) (Wu et al., 2019; Velázquez-Suárez et al., 2021).

### Statistical analysis

Statistical analysis of data was performed using the SPSS 22.0 software (IBM Corporation, Armonk, NY, USA). The data were compared and analyzed using the chi-square or Fisher’s exact test. All statistical tests were two-tailed. Data with *p* < 0.05 were considered statistically significant, and those with *p* < 0.01 were considered of high statistical significance.

## Results

### Basic information and burn severity grading

In total, ninety burn patients (60 males and 30 females, aged 1–94 years, mean age: 29.85 ± 17.84 years) were included in this study; 34, 27, 5, and 24 burn patients were categorized as mild, moderate, severe, and extra severe burn cases, respectively.

### MLST, *spa*, *agr*, and SCC*mec* typing

**Figure 1:**
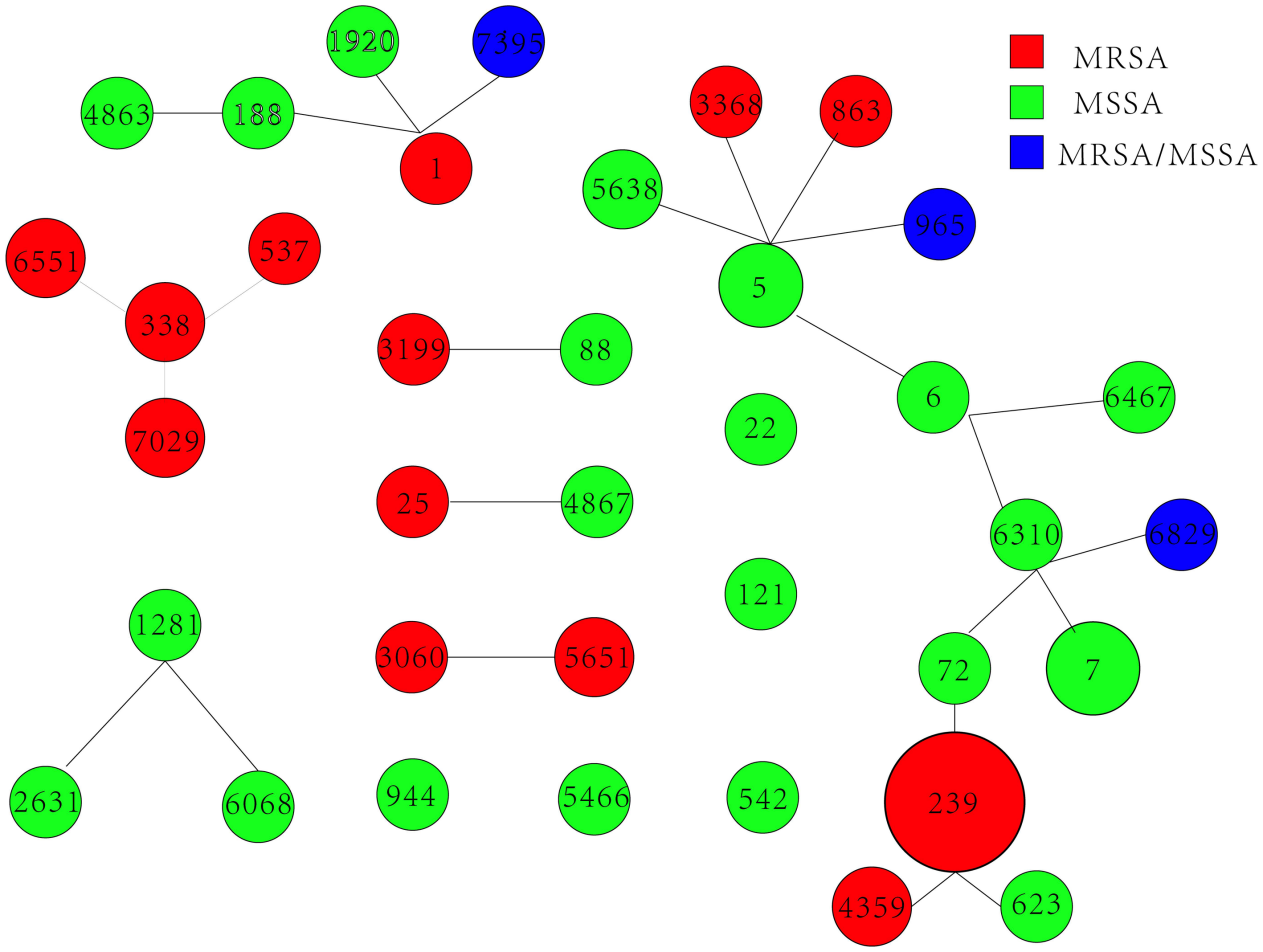
Distribution of STs in the clonal complexes. eBURST graph generated from the MLST data shows the relationships among the 90 *S. aureus* isolates. Each number denotes an MLST. STs linked by a line belong to the same cluster, and the circular area indicates the prevalence of those STs in the MLST data.

**Figure 2:**
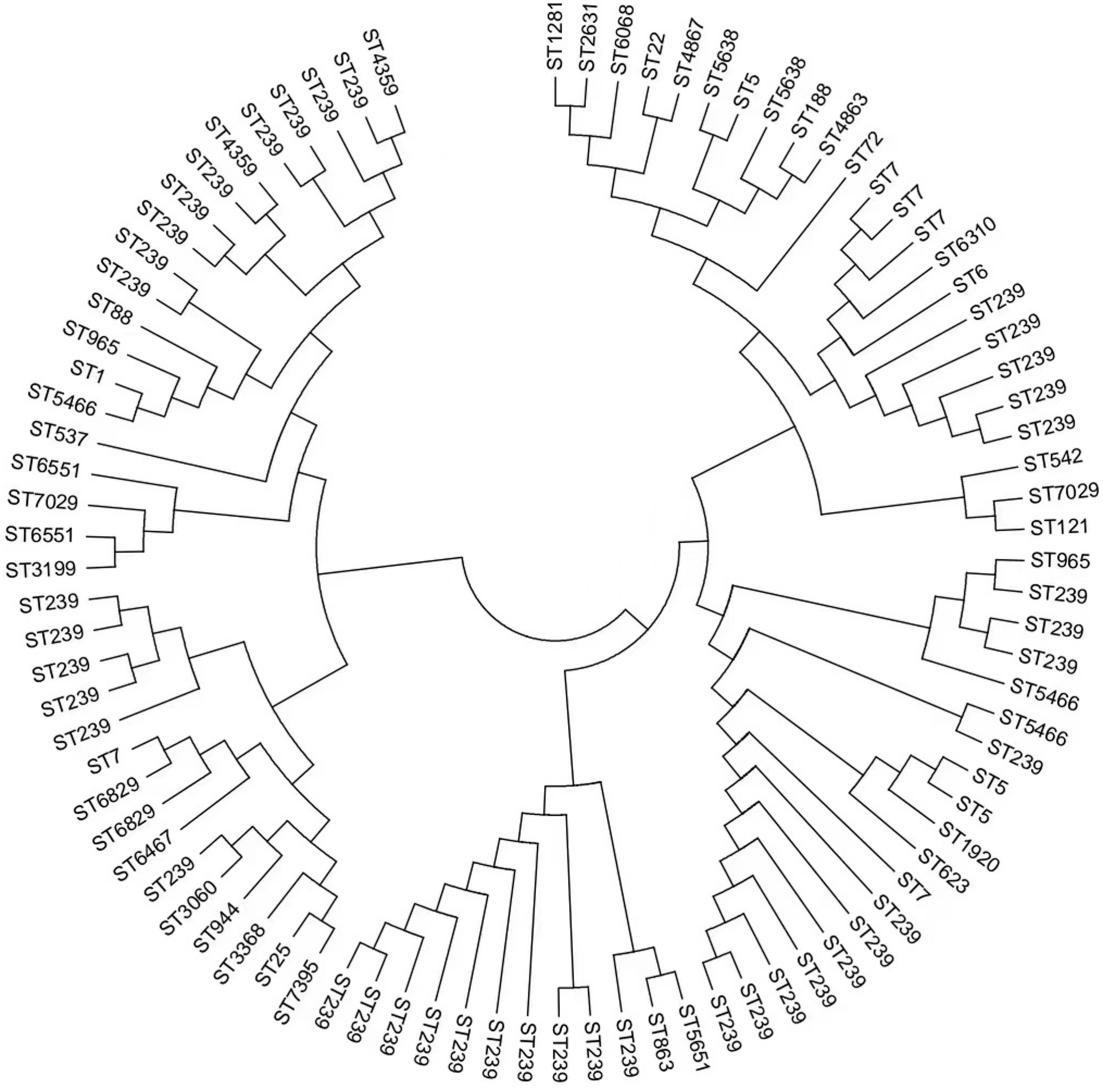
Neighbor-joining phylogeny of the 90 *S. aureus* isolates. The neighbor-joining (NJ) tree was constructed using the concatenated sequences of the multilocus sequence typing (MLST) scheme.

We obtained 90 *S. aureus* isolates, including 59 MRSA and 31 MSSA, from 5 hospitals in Fujian province, China. Concerning MRSA, 16 distinct STs were identified, and ST239 (66.1%, 39/59) was the most common MRSA. MSSA showed higher diversity than MRSA; ST 7 (16.1%, 5/31) was the most prevalent MSSA. ST7395, ST965, and ST6829 were found in both MRSA and MSSA. eBURST investigation identified 37 STs belonging to 7 groups and 5 singletons (Table 1 and Figure 1). Furthermore, we generated phylogenetic trees of STs to investigate their heterogeneity. STs were clustered into several clades, indicating their high diversity. Different independent branches belonging to ST239 indicated that this subtype originated from different evolutionary clades (Figure 2).

**Table 1:** Molecular characteristics of the 90 *Staphylococcus aureus* isolates collected in this study.

Group	CC	MLST	*spa*	*agr*	SCC*mec*	Ratio; *n* (%)
MRSA (*n* = 59)	CC8	ST 239	t030	I	III	34 (57.6%)
		ST 239	t030	NT	III	3 (5.1%)
		ST 239	t030	I	NT	1 (1.7%)
		ST239	t037	I	NT	1 (1.7%)
		ST 4359	t030	I	III	2 (3.4%)
	CC1	ST 1	t127	III	III	1 (1.7%)
		ST 7395	t114	NT	NT	1 (1.7%)
	CC45	ST 5651	t116	I	IVa	2 (3.4%)
		ST 3060	t1714	I	IVa	1 (1.7%)
	CC5	ST 965	t063	I	NT	1 (1.7%)
		ST 3368	t1084	NT	II	1 (1.7%)
		ST 863	t1084	NT	II	1 (1.7%)
	CC59	ST 338	t437	I	III	2 (3.4%)
		ST 6551	t441	I	III	1 (1.7%)
		ST 6551	t437	I	IVa	1 (1.7%)
		ST 537	t437	I	IVa	1 (1.7%)
	CC25	ST 25	t081	I	NT	1 (1.7%)
	Singleton	ST 7029	t437	III	IVa	1 (1.7%)
	Singleton	ST 7029	t13774	I	IVa	1 (1.7%)
	Singleton	ST 3199	t6497	III	V	1 (1.7%)
	Singleton	ST 6829	t091	I	NT	1 (1.7%)
MSSA (*n* = 31)	CC7	ST 7	t091	I	/	5 (16.1%)
	CC5	ST 5638	t686	II	/	1 (3.2%)
		ST 5638	NT	NT	/	1 (3.2%)
		ST 5	t954	NT	/	1 (3.2%)
			t002	NT	/	1 (3.2%)
			t548	NT	/	1 (3.2%)
		ST 6467	t701	I	/	1 (3.2%)
		ST 6	t701	I	/	1 (3.2%)
		ST 965	t062	NT	/	1 (3.2%)
	CC1	ST 1920	t286	III	/	1 (3.2%)
		ST 7395	t127	III	/	1 (3.2%)
		ST 4863	t189	I	/	1 (3.2%)
		ST 188	t189	I	/	1 (3.2%)
		ST 5466	NT	NT	/	1 (3.2%)
	CC8	ST 623	t17950	I	/	1 (3.2%)
		ST 72	t148	I	/	1 (3.2%)
	CC121	ST 121	t2091	I	/	1 (3.2%)
	CC22	ST 22	t309	III	/	1 (3.2%)
	CC30	ST 542	t338	III	/	1 (3.2%)
	CC2	ST 88	t3155	III	/	1 (3.2%)
	CC944	ST 944	t364	I	/	1 (3.2%)
	Singleton	ST 2631	t650	I	/	1 (3.2%)
	Singleton	ST 4867	t524	I	/	1 (3.2%)
	Singleton	ST 6310	t796	NT	/	1 (3.2%)
	Singleton	ST 6068	t20720	I	/	1 (3.2%)
	Singleton	ST 6829	t091	I	/	1 (3.2%)
	Singleton	ST 1281	t164	III	/	1 (3.2%)

The *spa* typing classified 59 MRSA isolates into 14 types; t030 was the most frequent type (67.8%, 40/59), followed by t437 (8.47%, 5/59). Among the 31 MSSA isolates, 22 genotypes were found, and t091 (19.4%,6/31) was the most present. Notably, we identified a new type, t20720, which has strong biofilm-forming ability and contains genes *pvl*, *lukED*, *lukAB, hlgCB, lukED, lukAB, hlgCB*, *icaA*, *icaB*, *icaC*, *icaD,* and *icaR*. Concerning *agr* typing, we identified three types: *agr*I (74.4%,67/90) was the most common, followed by *agr*III (10%,9/90). Interestingly, *agr*I and *agr*III exhibited a strong correlation with burn severity (*p* < 0.05) (Table 2).

**Table 2:** Distribution of virulence gene-positive strains in each burn grade.

Toxicity factors	Mild burn	Moderate burn	Severe burn	Extra severe burn	*R*	Value of *p*
*pvl* (*n* = 45)	21 (46.7%)	12 (26.7%)	2 (4.4%)	10 (22.2%)	0.172	0.105
*lukED* (*n* = 73)	28 (38.4%)	22 (30.1%)	3 (4.1%)	20 (27.4%)	0.016	0.884
*lukAB* (*n* = 56)	28 (50.0%)	18 (32.1%)	2 (3.6%)	8 (14.3%)	0.407	0.000
*hlgBC* (*n* = 24)	13 (54.2%)	9 (37.5%)	1 (4.2%)	1 (4.2%)	0.296	0.005
*eta* (*n* = 4)	1 (25.0%)	2 (50.0%)	0	1 (25.0%)	−0.022	0.838
*icaA* (*n* = 90)	34 (37.8%)	27 (30.0%)	5 (5.6%)	24 (26.7%)	/	/
*icaB* (*n* = 87)	33 (37.9%)	26 (29.9%)	5 (5.7%)	23 (26.4%)	0.019	0.860
*icaC* (*n* = 87)	34 (39.1%)	25 (28.7%)	5 (5.7%)	23 (26.4%)	0.095	0.371
*icaD* (*n* = 90)	34 (37.8%)	27 (30.0%)	5 (5.6%)	24 (26.7%)	/	/
*icaR* (*n* = 86)	31 (36.0%)	27 (31.4%)	5 (5.8%)	23 (26.7%)	−0.112	0.295
Biofilm (*n* = 72)	25 (34.7%)	21 (29.2%)	4 (5.6%)	22 (30.6%)	0.172	0.105
*agr* I (*n* = 67)	21 (31.3%)	21 (31.3%)	3 (4.5%)	22 (32.8%)	−0.254	0.016
*agr* II (*n* = 1)	1 (100%)	0	0	0	NA	NA
*agr* III (*n* = 9)	8 (88.9%)	1 (11.1%)	0	0	−0.333	0.001

Among the 59 MRSA isolates, four SCC*mec* subtypes (II, III, Iva, and V) were found. The most common was subtype III (72.9%,43/59), followed by subtype IVa (11.9%,7/59), and then subtype II (3.4%, 2/59). Nine isolates were classified as NT for SCC*mec* typing. All 59 MRSA isolates were *mec*A-positive. Based on combined (MLST, *spa*, *agr,* and SCC*mec*)typing, the major molecular clone of MRSA was CC8-ST239-t030-*agr*I-SCC*mec*III (55.9%,33/59), while the major molecular clone of MSSA was CC7-ST7-t091-*agr*I (16.13%,5/31) (Table 1).

### Antimicrobial susceptibility of ST239 and non-ST239

ST239 is a widespread burn wound infection in Fujian, China. Therefore, we compared its antimicrobial susceptibility with non-ST239. Statistical analysis revealed that ST239 isolates had significantly higher resistance than non-ST239 isolates for OXA, ERY, CLI, TET, GEN, CIP, LEV, and RIF antibiotics (*p* < 0.05). No *S. aureus* isolate was resistant to QD, VAN, LZD, and TCG antibiotics (Table 3).

**Table 3:** Susceptibility analysis of ST239 and non-ST239.

Drugs	ST239 (*n* = 39)			non ST239 (*n* = 51)			*p-value*	Susceptible	Intermediate	Resistant	Susceptible	Intermediate	Resistant
PEN	/	/	100%	2.00%	/	98.0%	1.000
OXA	/	/	100%	60.8%	/	39.2%	0.000
ERY	2.6%		97.4%	35.3%	2.0%	62.7%	0.000
CLI	5.1%		94.9%	39.2%	/	60.8%	0.000
TET	2.6%		97.4%	60.8%	/	39.2%	0.000
GEN	2.6%		97.4%	78.4%	/	21.6%	0.000
CIP	/	2.6%	97.4%	82.4%	/	17.6%	0.000
LEV	2.6%	33.3%	64.1%	76.5%	2.0%	21.5%	0.000
RIF	2.5%	2.5%	95.0%	90.2%	3.9%	5.9%	0.000
SXT	97.4%		2.6%	88.2%		11.8%	0.106
QD	100%	/	/	100%	/	/	/
LZD	100%	/	/	100%	/	/	/
TCG	100%	/	/	100%	/	/	/
VAN	100%	/	/	100%	/	/	/

PEN, penicillin; OXA, oxacillin; ERY, erythromycin; CLI, clindamycin; TET, tetracycline; GEN, gentamicin; CIP, ciprofloxacin; LEV, levofloxacin; RIF, rifampicin; SXT, trimethoprim/sulfamethoxazole; QD, quinupristin/dalfopristin; LZD, linezolid; TCG, tigecycline; and VAN, vancomycin.

### Biofilm formation ability

As quantified by TCD, of the 90 strains of *S. aureus* studied, 80% (72/90) of isolates were biofilm producers in varying degrees. A total of 37.8% (34/90) of the strains had a weak ability to form biofilms, 26.7%(24/90) had moderate ability, and 15.6%(14/90) strong ability. No significant difference between MRSA and MSSA (*p* > 0.05). The result of biofilm formation is shown in Table 4.

**Table 4:** Biofilm formation ability of *Staphylococcus aureus* from burn wound infections.

	Total positive rate	Number of strains with biofilm-forming capacity (%)				Value of *p*	Strong	Moderate	Weak	Negative
MRSA	50 (84.8%)	8 (13.6%)	15 (25.4%)	27 (45.8%)	9 (15.3%)	0.147
MSSA	22 (70.9%)	6 (19.4%)	9 (29.0%)	7 (22.6%)	9 (29.0%)	

### Virulence gene profiles

Over 90% of burn wound *S. aureus* isolates had *icaA* (100%), *icaD* (100%), *icaB* (96.7%), *icaC* (96.7%), and *icaR* (95.6%) virulence genes. Over 50% of burn wound *S. aureus* isolates had *lukED* (81.1%), *lukAB* (62.2%), and *pvl* (50.0%) virulence genes. Meanwhile, *hlgBC* (26.7%) and *eta* (4.4%) were the least prevalent virulence genes among burn wound *S. aureus* isolates. None of the isolates carried *lukMF′*and *etb* genes. MRSA isolates were less likely, compared with MSSA isolates, to carry *pvl*, *lukAB,* and *hlgBC* (*p* < 0.05) (Table 5).

**Table 5:** The frequency of virulence genes among *Staphylococcus aureus* isolates.

	Total population; *n* (%)	MRSA; *n* (%)	MSSA; *n* (%)	Value of *p*
Leukotoxin				
*pvl*	45 (50.0%)	23 (39.0%)	22 (71.0%)	0.040
*lukED*	73 (81.1%)	46 (78.0%)	27 (87.1%)	0.293
*lukAB*	56 (62.2%)	27 (45.8%)	29 (93.5%)	0.000
*hlgBC*	24 (26.7%)	5 (8.5%)	19 (61.3%)	0.000
*lukMF′*	0	0	0	/
Epidermal exfoliation toxin				
*eta*	4 (4.4%)	1 (1.7%)	3 (9.7%)	0.116
*etb*	0	0	0	/
Ica operon				
*icaA*	90 (100%)	59 (100%)	31 (100%)	/
*icaB*	87 (96.7%)	57 (96.6%)	30 (96.8%)	1.000
*icaC*	87 (96.7%)	56 (94.9%)	31 (100%)	0.549
*icaD*	90 (100%)	59 (100%)	31 (100%)	/
*icaR*	86 (95.6%)	57 (96.6%)	29 (93.5%)	0.606

*lukAB* and *hlgBC* genes showed a good correlation with BSG (*p* < 0.05). However, *pvl*, *lukED*, *eta*, *icaA*, *icaB*, *icaC*, *icaD*, *icaR*, and biofilm formation ability showed no correlation with BSG (Table 2).The distribution of molecular types in *S. aureus* isolates from burn wound infections is shown in Table 6.

**Table 6:** Distribution of molecular types in *Staphylococcus aureus* isolates from burn wound infections.

Group	CC (No;%)	MLST (No;%)	*spa* (No;%)	*agr* (No;%)	SCC*mec* (No;%)	Toxin profile (No;%)	Biofilm (No; %)	Antibiotic resistance profile (No; %)
MRSA (*n* = 59)	CC8 (41;69.5)	ST239 (39;95.1), ST 4359 (2;4.9)	t030 (40;97.6), t037 (1;2.4)	I (40;97.6), NT (1;2.4)	III (39;95.1), NT (2;4.9)	*icaA*, *icaD* (39;95.1), *icaB*, *icaC*, *icaR* (37;90.2), *lukED* (32;78.0), *lukAB* (16;39.0), *pvl* (14;34.1), *hlgBC* (3;7.3)	Absent (5;12.2), weak (18;43.9), moderate (13;31.7), strong (5;12.2)	PEN, OXA (41;100), GEN, CIP, TET, ERY (40;97.6), CLI, RIF (39;95.1), LEV (27;65.9), SXT (1;2.4)
	CC1 (2;3.4)	ST 1 (1;50), ST 7395 (1;50)	t127 (1;50), t114 (1;50)	III (1;50), NT (1;50)	III (1;50), NT (1;50)	*lukED*, *lukAB*, *icaA*, *icaB*, *icaC*, *icaD*, *icaR* (2;100)	Weak (1;50), strong (1;50)	PEN, OXAGEN, ERY (2;100), CLI, LEV, CIP, TET, SXT, RIF (1;50)
	CC45 (3;5.1)	ST 5651 (2;66.7), ST 3060 (1;33.3)	t116 (2;66.7), t1714 (1;33.3)	I (3;100)	IVa (3;100)	*icaA*, *icaB*, *icaD*, *icaR* (3;100), *icaC*, *pvl* (2;66.7), *lukED*, *eta* (1; 33.3)	Absent (2;66.7), weak (1;33.3)	PEN, OXA, ERY, CLI (3;100)
	CC5 (3;5.1)	ST 965 (1;33.3), ST 3368 (1;33.3), ST 863 (1;33.3)	t1084 (2;66.7), t063 (1;33.3)	I (1;33.3), NT (2;66.7)	II (2;66.7), NT (1;33.3)	*icaA*, *icaB*, *icaC*, *icaD, icaR*, *lukED* (3;100), *lukAB*, *pvl* (1;33.3)	Weak (2;66.7), moderate (1;33.3)	PEN, OXA, GEN, CIP, ERY, CLI, TET (3;100), LEV (2;66.7), SXT (1;33.3)
	CC59 (5;8.5)	ST 6551 (2;40.0), ST 338 (2;40.0), ST 537 (1;20.0)	t437 (4;80.0), t441 (1;20.0)	I (5;100)	III (3;60.0), IVa (2;40.0)	*icaA*, *icaB*, *icaC*, *icaD icaR* (5;100), *pvl*, *lukAB* (4;80.0), *lukED* (3;60.0)	Absent (1;20), weak (3;60), moderate (1;20)	PEN, OXA, ERY, CLI (5;100)
	CC25 (1;1.7)	ST 25 (1;100)	t081 (1;100)	I (1;100)	NT (1;100)	*icaA*, *icaB*, *icaC*, *icaD* *icaR*, *lukED*, *lukAB* (1; 100)	weak (1;100)	PEN; OXA (1;100)
	Singleton (4;6.8)	ST 7029 (2;50.0), ST 3199 (1;25.0), ST 6829 (1;25.0)	t13774 (1;25.0), t437 (1;25.0), t6497 (1;25.0), t091 (1;25.0)	I (2;50.0), III (2;50.0)	IVa (2;50.0), V (1;25.0), NT (1;25.0)	*icaA*, *icaB*, *icaC*, *icaD*, *icaR* (4;100), *lukAB* (3;75.0), *pvl*, *lukED* (2;50.0), *hlgBC* (1;25.0)	absent (1; 25.0), moderate (1;25.0), strong (2;50.0)	PEN, OXA (4;100), TET, ERY, CLI (3;75.0), LEV (1;25.0)
MSSA (*n* = 31)	CC7 (5;16.1)	ST 7 (5;100)	t091 (5;100)	I (5;100)	/	*icaA*, *icaC*, *icaD*, *icaR* *lukED* (5;100), *icaB*, *lukAB*, *pvl*, *hlgBC* (4;80.0), *eta* (1;20.0)	moderate (2;40.0), strong (3;60.0)	PEN (5;100), TET (4;80.0)
	CC5 (8;25.8)	ST 5 (3;37.5), ST 5638 (2;25.0), ST 6467 (1;12.5), ST 6 (1;12.5), ST 965 (1;12.5)	t701 (2;25.0), t686 (1;12.5), t954 (1;12.5), t002 (1;12.5), t548 (1;12.5), t062 (1;12.5), NT (1;12.5)	II (1;12.5), I (2;25.0), NT (5;62.5)	/	*icaA*, *icaB*, *icaC*, *icaD* *icaR* (8;100), *lukAB*, *lukED*, *hlgBC* (7;87.5), *pvl* (5;62.5), *eta* (1;12.5)	Absent (5;62.5), moderate (1;12.5), strong (2;25.0)	PEN (8;100), ERY (4;50.0), CLI (3;37.5), SXT, CIP, LEV (2;25.0), GEN (1;12.5)
	CC1 (5;16.1)	ST1920 (1;20.0), ST 7395 (1;20.0), ST 4863 (1;20.0), ST 188 (1;20.0), ST 5466 (1;20.0)	t189 (2;40.0), t127 (1;20.0), t286 (1;20.0), NT (1;20.0)	I (2;40.0), III (2;40.0), NT (1,20.0)	/	*icaA*, *icaB*, *icaC*, *icaD* *pvl*, *lukAB* (5;100), *lukED*, *icaR* (4;80.0), *hlgBC* (3;60.0)	Absent (1;20.0), weak (3;60.0), moderate (1;20.0)	PEN (5;100), ERY, CLI (1;20.0)
	CC8 (2;6.5)	ST 623 (1;50.0), ST 72 (1;50.0)	t17950 (1;50.0), t148 (1;50.0)	I (2;100)	/	*icaA*, *icaB*, *icaC*, *icaD* *icaR*, *lukED*, *lukAB* (2;100), *pvl, hlgBC* (1;50.0)	Absent (2;100)	PEN, GEN, ERY, CLI, LEV (1;50.0)
	CC121 (1;3.2)	ST 121 (1;100)	t2091 (1;100)	I (1;100)	/	*icaA*, *icaB*, *icaC*, *icaD*, *icaR*, *lukAB*, *lukED*, *hlgBC*, *pvl*, *eta* (1;100)	Moderate (1;100)	PEN, ERY, CLI (1;100)
	CC22 (1;3.2)	ST 22 (1;100)	t309 (1;100)	III (1;100)	/	*icaA*, *icaB*, *icaC*, *icaD*, *icaR*, *lukAB*, *lukED*, *hlgBC*, *pvl* (1;100)	Weak (1;100)	PEN, ERY, CLI (1;100)
	CC30 (1;3.2)	ST 542 (1;100)	t338 (1;100)	III (1;100)	/	*icaA*, *icaB*, *icaC*, *icaD*, *icaR*, *hlgBC* (1;100)	Moderate (1;100)	PEN, ERY CLI, LEV (1;100)
	CC2 (1;3.2)	ST 88 (1;100)	t3155 (1;100)	III (1;100)	/	*icaA*, *icaB*, *icaC*, *icaD*, *icaR*, *lukAB*, *lukED* (1; 100)	Moderate (1;100)	PEN, TET (1;100)
	CC944 (1;3.2)	ST 944 (1;100)	t364 (1;100)	I (1;100)	/	*icaA*, *icaB*, *icaC*, *icaD*, *icaR*, *lukED*, *hlgBC* (1;100)	Weak (1;100)	PEN, ERY, CLI (1;100)
	Singleton (6;19.4)	ST 2631 (1;16.7), ST 4867 (1;16.7), ST 6310 (1;16.7), ST 6068 (1;16.7), ST 6829 (1;16.7), ST 1281 (1;16.7)	t650 (1;16.7), t524 (1;16.7), t796 (1;16.7), t20720 (1;16.7), t091 (1;16.7), t164 (1;16.7)	I (4;66.7), III (1;16.7), NT (1;16.7)	/	*lukAB*, *lukED* (6;100), *pvl*, *icaR* (5;83.3), *hlgBC* (3;50.0), *icaA*, *icaB*, *icaC*, *icaD* (1;16.7)	Absent (1;16.7), strong (1;16.7) weak (2;33.3), moderate (2;33.3)	ERY, CLI (4;66.7), SXT, GEN (2;33.3), CIP, TET LEV, PEN (1;16.7)

## Discussion

Here, we conducted a multicenter prospective study to determine the molecular epidemiology and antimicrobial susceptibility of *S. aureus* isolates from burn wound infections. This study presents an extensive collection of 90 *S. aureus* isolates collected from five different areas in Fujian, China, covering a substantial geographical zone of the city. This is so far a major multicenter study of burn wound infections from Fujian, China.

*Staphylococcus aureus* burn wound infection has a high incidence rate and mortality (Tong et al., 2015). Skin, the first line of defense against microbial invasion, becomes more prone to infection after burns. *S. aureus* secretes thick exudates during wound infections, causing inflammation. Tissue necrosis and graft dissolution occur in the late residual of wound burns. Without proper treatment, the wound continues to deteriorate causing severe complications, such as systemic infection or sepsis, leading to high mortality. Previous studies mainly focused on MRSA isolates (Rodrigues et al., 2013; Amissah et al., 2017). However, MSSA is an important opportunistic pathogen in burn wound infections (Dad et al., 2022). Our study focused on *S. aureus* infections in burn wounds, including MRSA and MSSA. Our result showed that the biofilm formation ability of MSSA was the same as that of MRSA (*p* > 0.05). Moreover, MSSA has a higher toxicity than MRSA. MRSA was less likely, compared with MSSA, to carry *pvl*, *lukAB*, and *hlgBC* (*p* < 0.05). These results suggest that MSSA is equally important in burn centers as MRSA.

The molecular epidemiology of *S. aureus* varies with the types of wound infections. For instance, ST88 and ST152 were the most abundant STs from chronically infected wounds (Wolters et al., 2020). ST59, ST8, and ST45 were the three leading MRSA isolates from skin and soft tissue infections (SSTIs) (Lee et al., 2022). ST30 was the most frequent clonal isolate from wound-related infections in Tehran, Iran (Goudarzi et al., 2019). In this study, CC8-ST239-t030-*agr*I-SCC*mec* III was the main clone of MRSA isolates. The possible reason for the spread of ST239 in burn wound infections could be its multidrug resistance, enabling its survival and spread in hospitals (Tong et al., 2015). Antimicrobial susceptibility tests showed high resistance of ST239 against OXA, ERY, CLI, TET, GEN, CIP, LEV, and RIF antibiotics (*p* < 0.05). The burn wound provides an ideal environment for the growth and reproduction of ST239 (Keen et al., 2010). A previous study showed that CC8-ST239-t030-*agr*I-SCC*mec*III was the most common clone in burn bloodstream infection (Liu et al., 2018). This highlights the importance of molecular surveillance in burn wound patients for timely and effective control of bloodstream infection.

SCC*mec* types can be identified between hospital-acquired methicillin-resistant *S. aureus* (HA-MRSA) and community-acquired methicillin-resistant *S. aureus* (CA-MRSA). HA-MRSA usually carries SCC*mec* I, II, or III, whereas CA-MRSA usually carries SCC*mec* IV or V (Cocchi et al., 2011). SCC*mec* IV and SCC*mec*V types were generally observed in community-acquired infections, but those strains were detected in this study and were acquired in hospitals. The result suggested that CA-MRSA-based clones may have successfully invaded hospital facilities. The molecular characteristics between CA-MRSA and HA-MRSA are blurring, consistent with previous study (Yu et al., 2022). The increased spread of CA-MRSA in the hospital environment may be related to the smaller size of SCC*mec*IVa and SCC*me*cV genes compared to other *S. aureus* strains (Zhang et al., 2005).

Our analysis also highlights the growing public health threat from livestock-associated methicillin-resistant *S. aureus* (LA-MRSA) clones. Here, we identified CC1-ST1-t127 carrying *lukED*, *lukAB*, and *icaABCDR* genes in MRSA isolates. Notably, CC1-ST1-t127 is one of the major LA-MRSA lineages and has been associated with pigs across European countries (Merialdi et al., 2019).^.^ There have been reports of human CC1-ST1-t127 infections. CC1-ST1-t127 was the sixth most prevalent clone isolated from human invasive infections in Europe (Grundmann et al., 2010) and the most common clone isolated from nursing homes in Shanghai, China (He et al., 2021). It was the fourth most prevalent MSSA isolate from a children’s hospital in Beijing, China (Wu et al., 2010). A previous study showed that about 77–86% of people with occupational contact with pigs carried LA-MRSA in their nasal cavities (Cuny et al., 2015). Fujian, situated on the southeast coast of China, is a leading modern characteristic agricultural production base, with livestock and poultry industries being its famous sectors. This may promote the spread of LA-MRSA in the Fujian region. Farmers and veterinarians who have contact with livestock are advised to undergo MRSA colonization screening upon hospitalization to prevent the clinical transmission of LA-MRSA.

MSSA has been shown to have higher genetic diversity and broader geographic distribution than MRSA (Wang et al., 2018). In this study, 24 different STs were identified from 31 MSSA isolates from burn wound infections, indicating their higher genetic diversity. The molecular signature of MSSA strains was CC7-ST7-t091-*agr*I. ST7 is a frequently seen MSSA type associated with foodborne illness (Guo et al., 2023). After the COVID-19 pandemic, ST7 isolates of *S. aureus* have become very prevalent in Wuhan, China. Notably, ST7 has strong abilities to acquire the SCC*mec* element and virulence genes under the pressure of disinfectant agents (Gu et al., 2023). All of this suggests that it is crucial to enhance the monitoring of ST7 variants of *S. aureus*. This study also identified ST22 strains of *S. aureus* that harbor *pvl* genes in MSSA isolates. Notably, ST22 was found to be the main MRSA clone in European countries, Australia, New Zealand, and Singapore^[38].^ A unique ST22 MRSA clone carrying the *pvl* and toxic shock syndrome toxin-1-encoding genes, named ST22-PT, was reported recently (Yamaguchi et al., 2022) and has sporadically been isolated in several countries (Boswihi et al., 2022). ST22-PT is highly virulent and causes fatal necrotizing pneumonia and sepsis (Uda et al., 2020). MSSA can become MRSA by acquiring SCC*mec* (Hayakawa et al., 2020); therefore, it is important to prevent the spread of ST22-*pvl* MSSA to MRSA.

*S. aureus* secretes a variety of virulence factors, which provide protection from the host’s innate immune response through various mechanisms and promote infection (Kim et al., 2012). *pvl* causes cell death and inflammation by acting on the cell membranes of macrophages, monocytes, and polymorphonuclear cells (Shallcross et al., 2013). *pvl* has high toxicity and a high mortality rate (Leistner et al., 2022). *pvl* has been associated with community-acquired staphylococcal infections, particularly those caused by CA-MRSA (Vandenesch et al., 2003). However, *pvl* was detected in hospital-acquired infections in previous studies (Benoit et al., 2008; Zhao et al., 2022), similar to ours. About 50% of the strains in our study carried the *pvl* gene. However, there have been differences in the detection rates of *pvl* reported from different provinces in China: Guangdong (27.2%) (Liang et al., 2019), Hainan (47.6%) (Li et al., 2019), Yunnan (92.4%) (Ma et al., 2022), Heilongjiang (13.3%) (Yang et al., 2021). Possibly, *S. aureus* strains gain the *pvl* virulence gene through the bacteriophage lysogenic transformation indicating the horizontal transmission of this gene (Monecke et al., 2012). In case of potential *pvl* infection, personal protective equipment should be used, and isolation should be enforced to prevent the spreading of that bacterial strain.

The *ica* locus (*ica*ADBC) encodes the enzyme *N*-acetyl-glucosaminyltranferase, which participates in the production of polysaccharide intercellular adhesion (PIA), facilitating the biofilm formation of *S. aureus* (Cue et al., 2012). A previous study has reported differences in the detection rate of *ica*ADBC in the biofilm formation process of *S. aureus,* and the absence of a certain *ica*ADBC may not necessarily affect the biofilm formation (Ghaioumy et al., 2021). This also explains the slightly higher detection rates of *ica*A and *ica*D (both 100%) than those of *ica*B and *ica*C (both 96.7%) in this study. Possibly, *S. aureus* can form biofilms through cell wall anchoring protein without PIA (Schneewind and Sortases, 2019).

Besides, there are some other notable findings from this study. Firstly, ST7395, ST965, and ST6829 were found in MRSA and MSSA isolates. The result indicated that some MSSA strains provided a stable genomic environment for integrating SCC*mec* elements, which helped transfer between isolates (Zhu et al., 2022). Secondly, consistent with previous studies (Javdan et al., 2019), *agr*I and *agr*III were related to the severity of burns. However, the correlation reasons were not clear and demanded further investigation. Thirdly, we found a new *spa* type t20720 of *S. aureus* in MSSA, which has strong biofilm formation ability and carries the *pvl*, *lukED*, *lukAB*, *hlgCB*, and *icaABCDR* virulence genes. This strain should be closely monitored.

## Conclusion

In conclusion, CC8-ST239-t030-*agr*I-SCC*mec*III and CC7-ST-7-t091-*agr*I were the prevalent molecular signature of MRSA and MSSA isolates from burn wound infections in Fujian, China, respectively. Virulence genes were widely distributed among *S. aureus,* and these strains could easily form biofilms. ST239 exhibited high drug resistance, and the newly identified *spa*-t20720 isolate requires special clinical attention. Prevention and control measures must be implemented to reduce the spread of these highly virulent and resistant strains.

## Data availability statement

The raw data supporting the conclusions of this article will be made available by the authors, without undue reservation.

## Author contributions

BL and XH designed the study and obtained the funding. XH and SZ performed the experiments. DX and GZ performed the statistical analysis. XH wrote the manuscript. BL and XD contributed to manuscript revision. YC contributed the materials. All authors contributed to the article and approved the submitted version.

## Funding

This work was supported by the Science and Technology Plan Project of Quanzhou, Fujian (grant number: 2020N54s) and Fujian Research and Training Grants for Young and Middle-aged Leaders in Healthcare.

## Conflict of interest

The authors declare that the research was conducted in the absence of any commercial or financial relationships that could be construed as a potential conflict of interest.

## Publisher’s note

All claims expressed in this article are solely those of the authors and do not necessarily represent those of their affiliated organizations, or those of the publisher, the editors and the reviewers. Any product that may be evaluated in this article, or claim that may be made by its manufacturer, is not guaranteed or endorsed by the publisher.

## Footnotes

^1^ http://spaserver.ridom.de

^2^ http://saureus.mlst.net

## Abbreviations

CA-MRSA: Community-acquired methicillin-resistant *Staphylococcus aureus*
HA-MRSA: Hospital-acquired methicillin-resistant *Staphylococcus aureus*
LA-MRSA: Livestock-associated methicillin-resistant *Staphylococcus aureus*
CC: clonal complex
MLST: multilocus sequence typing
MRSA: methicillin-resistant *Staphylococcus aureus*
MSSA: methicillin-susceptible *Staphylococcus aureus*
*pvl*: Panton-Valentine Leukotoxin
*S. aureus*: *Staphylococcus aureus*
SCC*mec*: *Staphylococcus* chromosomal cassette *mec*
NT: non-typeable
