# Supplementary material for: Molecular typing and characterization of Staphylococcus aureus isolates from burn wound infections in Fujian, China
Source: Front Microbiol. 2023 Sep 20;14:1236497. doi: 10.3389/fmicb.2023.1236497 (PMC10547878; doi:10.3389/fmicb.2023.1236497)
Supplement: Supplementary file 1 [file Table_1.docx]

**Additional file 1**

**Molecular typing and characterization of *Staphylococcus aureus* isolates from burn wound infections in Fujian, China**

**Xiaolan Hong^1,2^, Shaobo Zhou^2^, Xubo Dai^2^, Dandan Xie^2^, Yuanyuan Cai^2^, Guimei Zhao^2^, BinLi^1^***

***Correspondence Prof. BinLi E-mail:leonlee307@hotmail.com**

**Additional file1** Sequences of primers used for PCR in this study

| Primer | Sequence (5′-3′) | |  | Amplicon size (bp) | Reference |
| --- | --- | --- | --- | --- | --- |
| **Leukotoxins gene** |  | | |  |  |
| *pvl-F* | ATCATTAGGTAAAATGTCTGGACATGATCCA  GCATCAACTGTATTGGATAGCAAAAGC GCCAATCCGTTATTAGAAAATGC  CCATAGAYGTAGCAACGGAT | | | 433 | [14] |
| *pvl -R* |  |  |  |  |  |
| *hlgBC-F* |  |  |  | 938 | [14] |
| *hlgBC-R* |  |  |  |  |  |
| *lukED-F* | TGAAAAAGGTTCAAAGTTGATACGAG | | | 269 | [14] |
| *lukED-R* | TGTATTCGATAGCAAAAGCAGTGCA | | |  |  |
| *lukAB-F* | TCACTTCTCCACCATACTTC | | | 636 | [14] |
| *lukAB-R* | TATCAGCAGCAACGACTC | | |  |  |
| *lukMFʹ-F* | TGGATGTTACCTATGCAACCTAC | | | 780 | [15] |
| *lukMFʹ-R* | GTTCGTTTCCATATAATGAATCACTAC | | |  |  |
| **Epidermal exfoliation toxin** | | | |  |  |
| *eta-F* | CGCTGCGGACATTCCTACATGG | | | 676 | [16] |
| *eta-R* | TACATGCCCGCCACTTGCTTGT | | |  |  |
| etb-F | CAGATAAAGAGCTTTATACACACATTAC | | | 612 | [16] |
| etb-R | AGTGAACTTATCTTTCTATTGAAAAACACTC | | |  |  |
| **Ica operon** |  | | |  |  |
| *icaA- F* | GAGGTAAAGCCAACGCACTC | | | 151 | [17] |
| *icaA- R* | | CCTGTAACCGCACCAAGTTT | |  |  |
| *icaB- F* | | ATACCGGCGACTGGGTTTAT | | 140 | [17] |
| *icaB- R* | | TTGCAAATCGTGGGTATGTGT | |  |  |
| *icaC- F* | | CTTGGGTATTTGCACGCATT | | 209 | [17] |
| *icaC- R* | | GCAATATCATGCCGACACCT | |  |  |
| *icaD- F* | | ACCCAACGCTAAAATCATCG | | 211 | [17] |
| *icaD- R* | | GCGAAAATGCCCATAGTTTC | |  |  |
| *icaR- F* | | ATCTA ATACGCCTGAGGA | | 205 | [17] |
| *icaR- R* | | TTCTTCCACTGCTCCAA | |  |  |
| **SCC*mec* typing** | |  | |  |  |
| *Type I-F* | | GCTTTAAAGAGTGTCGTTACAGG | | 613 | [14] |
| *Type I-R* | | GTTCTCTCATAGTATGACGTCC | |  |  |
| *Type II-F* | | CGTTGAAGATGATGAAGCG | | 398 | [14] |
| *Type II-R* | | CGAAATCAATGGTTAATGGACC | |  |  |
| *Type III-F* | | CCATATTGTGTACGATGCG | | 280 | [14] |
| *Type III-R* | | CCTTAGTTGTCGTAACAGATCG | |  |  |
| *TypeIVa-F* | | GCCTTATTCGAAGAAACCG | | 776 | [14] |
| *TypeIVa-R* | | CTACTCTTCTGAAAAGCGTCG | |  |  |
| *TypeIVb-F* | | TCTGGAATTACTTCAGCTGC | | 493 | [14] |
| *Type IVb-R* | | AAACAATATTGCTCTCCCTC | |  |  |
| *Type IVc-F* | | ACAATATTTGTATTATCGGAGAGC | | 200 | [14] |
| *Type IVc-R* | | TTGGTATGAGGTATTGCTGG | |  |  |
| *Type IVd-F* | | CTCAAAATACGGACCCCAATACA | | 881 | [14] |
| *Type IVd-R* | | TGCTCCAGTAATTGCTAAAG | |  |  |
| *Type V-F* | | GAACATTGTTACTTAAATGAGCG | | 325 | [14] |
| *Type V-R* | | TGAAAGTTGTACCCTTGACACC | |  |  |
| *mecA -F* | | GTGAAGATATACCAAGTGATT | | 147 | [14] |
| *mecA-R* | | ATGCGCTATAGATTGAAAGGAT | |  |  |
| **Agr typing** | |  | |  |  |
| *Pan* | | ATGCACATGGTGCACATGC | | / | [14] |
| *agr I* | | GTCACAAGTACTATAAGCTGCGAT | | 439 | [14] |
| *agr II* | | TATTACTAATTGAAAAGTGGCCATAGC | | 572 | [14] |
| *agr III* | | GTAATGTAATAGCTTGTATAATAATACCCAG | | 321 | [14] |
| *agr IV* | | CGATAATGCCGTAATACCCG | | 657 | [14] |
| **Spa typing** | |  | |  |  |
| *spa-1113-F* | | TAAAGACGATCCTTCGGTGAGC | | 300-400 | [13] |
| *spa-1514-R* | | CAGCAGTAGTGCCGTTTGCTT | |  |  |
| **MLST typing** | |  | |  |  |
| *arc-F* | | TTGATTCACCAGCGCGTATTGTC | | 456 | [14] |
| *arc-R* | | AGGTATCTGCTTCAATCAGCG | |  |  |
| *aro-F* | | ATCGGAAATCCTATTTCACATTC | | 456 | [14] |
| *aro-R* | | GGTGTTGTATTAATAACGATATC | |  |  |
| *glp-F* | | CTAGGAACTGCAATCTTAATCC | | 465 | [14] |
| *glp-R* | | TGGTAAAATCGCATGTCCAATTC | |  |  |
| *gmk-F* | | ATCGTTTTATCGGGACCATC | | 429 | [14] |
| *gmk-R* | | TCATTAACTACAACGTAATCGTA | |  |  |
| *pta-F* | | GTTAAAATCGTATTACCTGAAGG | | 474 | [14] |
| *pta-R* | | GACCCTTTTGTTGAAAAGCTTAA | |  |  |
| *tpi-F* | | TCGTTCATTCTGAACGTCGTGAA | | 402 | [14] |
| *tpi-R* | | TTTGCACCTTCTAACAATTGTAC | |  |  |
| *yqi-F* | | CAGCATACAGGACACCTATTGGC | | 516 | [14] |
| *yqi-R* | | CGTTGAGGAATC GATACTGGAAC | |  |  |
